# A clinical study exploring the prediction of microvascular invasion in hepatocellular carcinoma through the use of combined enhanced CT and MRI radiomics

**DOI:** 10.1371/journal.pone.0318232

**Published:** 2025-01-28

**Authors:** Jiangfa Li, Wenxiang Song, Jixue Li, Lv Cai, Zhao Jiang, Mengxiao Wei, Boming Nong, Meiyu Lai, Yiyi Jiang, Erbo Zhao, Liping Lei

**Affiliations:** 1 Department of Hepatobiliary and Pancreatic Surgery, Affiliated Hospital of Guilin Medical University, Guilin, Guangxi, China; 2 Key Laboratory of Early Prevention and Treatment for Regional High Frequency Tumor (Guangxi Medical University), Ministry of Education, Nanning, Guangxi, China; 3 Guangxi Key Laboratory of Early Prevention and Treatment for Regional High Frequency Tumor, Nanning, Guangxi, China; 4 Department of Information, Affiliated Hospital of Guilin Medical University, Guilin, Guangxi, China; 5 Department of Geriatric Medicine, the Affiliated Hospital of Guilin Medical University, Guilin, Guangxi, China; Kaohsiung Medical University Hospital, TAIWAN

## Abstract

**Objective:**

To develop a predictive model for microvascular invasion (MVI) in hepatocellular carcinoma (HCC) through radiomics analysis, integrating data from both enhanced computed tomography (CT) and magnetic resonance imaging (MRI).

**Methods:**

A retrospective analysis was conducted on 93 HCC patients who underwent partial hepatectomy. The gold standard for MVI was based on the histopathological diagnosis of the tissue. The 93 patients were randomly divided into training and validation groups in 7:3 ratio. The imaging data of patients, including CT and MRI, were collected and processed using 3D Slicer to delineate the region of interest (ROI) for each tumor. Radiomics features were extracted from CT and MRI of patients using Python. Lasso regression analysis was used to select optimal radiomics features for MVI in the training group. The optimal radiomics features of CT and MRI were selected to establish the prediction model. The predictive performance of the model was evaluated using the receiver operator characteristic curve (ROC), calibration curve, and decision curve analysis (DCA).

**Results:**

After univariate and multivariate analyses, it was found that tumor diameter was significantly different between the MVI positive and negative groups. After extracting 2153 imaging phenotyping features from the CT and MRI images of the 93 patients using Python, ten standardized coefficient non-zero imaging phenotyping features were finally determined by Lasso regression analysis in the CT and MRI images. A comprehensive predictive model with clinical variable and optimal radiomics features was established. The area under the curve (AUC) of the training group was 0.916 (95%CI: 0.843–1.000), sensitivity: 95.2%, specificity: 79.2%. In the validation group, the predictive model diagnosed MVI with AUC = 0.816 (95%CI: 0.642–0.990), sensitivity: 84.2%, and specificity: 75.0%.

**Conclusion:**

The joint model that integrated the optimal radiomics features with clinical variables has good diagnostic performance for MVI of HCC and specific clinical applicability.

## Introduction

Primary liver cancer is currently the fourth most common malignant tumor and the second leading cause of cancer-related death in China, posing a severe threat to public health [1]. Despite advancements in the diagnosis and treatment of hepatocellular carcinoma (HCC), patient prognosis remains poor due to low overall survival rates and high recurrence rates post-treatment [2]. Vascular invasion is a critical factor in HCC prognosis, particularly in contributing to tumor recurrence following hepatectomy and liver transplantation [3,4]. Microvascular invasion (MVI) is recognized as a risk factor for recurrence and metastasis after HCC treatment [4]. Research by Lin et al. indicates that increasing the resection margin width and tumor size during surgery can reduce postoperative recurrence rates in patients with MVI [5]. Therefore, preoperative prediction of MVI is crucial for assessing HCC prognosis, selecting appropriate surgical strategies, and developing effective treatments for recurrence and metastasis. Recent studies have focused on reliable preoperative MVI prediction methods, including imaging techniques, serum markers, and gene expression profiles [6–8]. The emergence of radiomics offers a promising approach for accurate disease assessment and personalized treatment planning [9]. Establishing a simple, rapid, and effective prediction model using enhanced CT and MRI images for preoperative MVI evaluation could significantly impact patient survival and treatment outcomes.

## Methods

### Patients

Clinical data of HCC patients who underwent partial hepatectomy at the Affiliated Hospital of Guilin Medical University between October 2018 and September 2023 were retrospectively analyzed. The clinical data were collected from October 2023 to January 2024. All patients included in the study had their diagnoses confirmed through surgical pathology. The study received approval from the Ethics Management Committee of the Affiliated Hospital of Guilin Medical University (2022YJSLL-02). Authors could not access to information that could identify individual participants after data collection.

### Inclusion criteria

Blood biochemical tests should be conducted within one week prior to the surgery. Additionally, abdominal enhanced CT and MRI scans should be performed within two weeks before the surgery. The patients did not undergo any preoperative anti-tumor or interventional therapies. The postoperative pathological report confirmed the presence or absence of MVI. In cases where MVI was not definitively diagnosed, pathological sections were re-evaluated to confirm the presence or absence of MVI. There was no history of other malignant tumors. The Child-Pugh classification was Grade A.

### Exclusion criteria

Patients diagnosed with HCC exhibited distant metastasis prior to undergoing surgery. Additionally, essential clinical data were found to be incomplete. There was major vascular invasion. The patient’s Performance Status score was more than 1.

### Pathological data

Postoperative pathological examinations categorized patients into two groups: MVI-positive and MVI-negative. This classification was based on the presence or absence of MVI identified in the surgical specimens.

### Imaging data

Abdominal-enhanced CT and MRI images taken within two weeks before surgery were collected. Enhanced CT scans were performed using Revolution CT scanners, including 256-row and 128-row spiral CT models from General Electric (GE, USA). Scans were conducted during the arterial phase (AP) and portal venous phase (PVP), covering the entire liver. MRI was conducted with a Siemens Magnetom Verio 3.0T MRI scanner (Siemens, Germany), utilizing gadolinium-

ethoxybenzyl-diethylenetriamine pentaacetic acid (Gd-EOB-DTPA) for contrast enhancement. Both routine scans and contrast-enhanced images with disodium gadolinite were obtained during the AP and PVP phases.

### Blood biochemical indexes

Blood biochemical examination reports were collected for HCC patients who met the inclusion criteria, with samples taken less than a week before surgery. On the day of admission, 5 ml of fasting venous blood from the right upper limb was collected at 6 a.m. The specimens were sent to the Clinical Laboratory of the Affiliated Hospital of Guilin Medical University for testing. The samples were centrifuged using a high-speed centrifuge at 4°C, 1500 rpm for 15 minutes, and the final test reports were generated accordingly.

### Image processing

The AP and PVP images of HCC patients which met the inclusion criteria were imported into 3D Slicer version 5.40 in DICOM format. The Fast T1-weighted sequence of AP and PVP was used in MRI. Separate cases for CT and MRI were created. A radiologist with five years of experience in abdominal disease diagnosis, unaware of the pathological results, manually delineated the region of interest (ROI) on both AP and PVP images for CT and MRI.

The ROI was carefully sketched to cover the entire tumor, including all bleeding or necrotic areas while avoiding peritumoral edema. In cases of blurred lesion edges, the maximum extent of the lesion was outlined. This process was repeated for the venous phase images to ensure consistency in the number, size, and position of ROI layers across different phases of imaging for the same patient. The final result was a volume of interest (VOI) that included comprehensive information on the HCC lesions.

**Fig 1:**
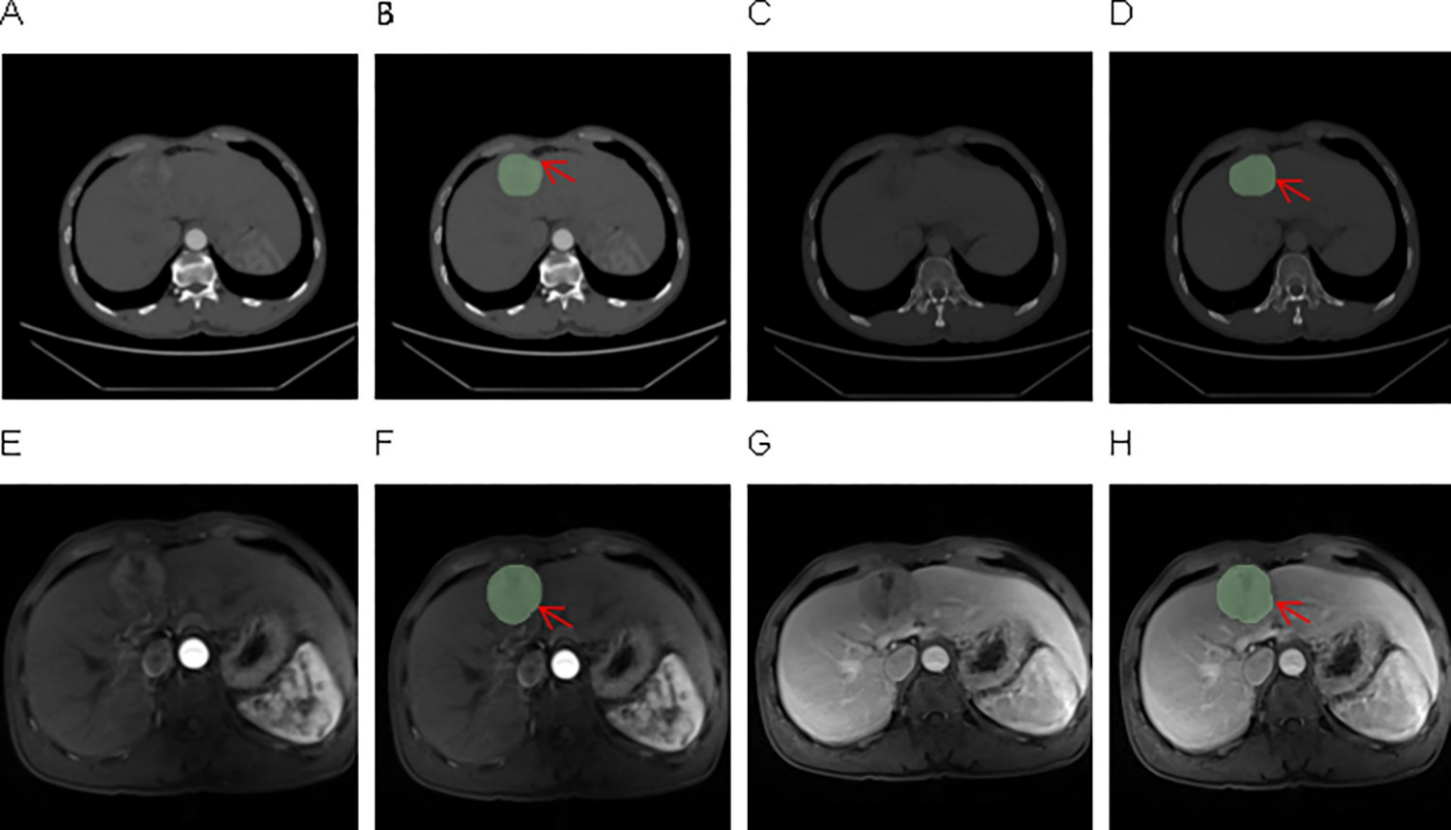
Extracted CT and MRI images and outlined ROI. (A) CT arterial phase images. (B) ROI sketched by CT in arterial phase. (C) CT venous phase images. (D) The ROI outlined in the venous phase of CT. (E) The arterial phase image of MRI. (F) ROI sketched by MRI in arterial phase. (G) The venous phase image of MRI. (H) The ROI outlined in the venous phase of MRI. ROI: The region of interest; CT: Computerized tomography; MRI: Nuclear magnetic resonance.

The segmented ROIs were then proofread layer by layer by a radiologist with over five years of experience in abdominal disease diagnosis to confirm the accuracy of the segmentation results. A diagram outlining the ROI was shown in Fig 1.

### Feature extraction and screening

The "Pyradiomics" package in Python (version 3.10.1) extracted features from the original and nine derived images. The derived images include:1.Wavelet filter, 2.Laplacian of Gaussian (LoG), 3. Square, 4. Square Root, 5.Logarithm6.Exponential, 7.Gradient, 8.Local Binary Pattern (2D), 9. Local Binary Pattern (3D). The Wavelet transform applied high-pass and low-pass filters to the original image, resulting in 8 combinations: HHH, HHL, HLL, HLH, LHL, LLH, and LLL, where H represents high-pass and L represents low-pass filtering. Features were extracted from both the original and derived images.

The extracted features included:1.First-order statistics, 2.Shape-based features,3.Gray Level Co-occurrence Matrix (GLCM),4. Gray Level Run Length Matrix (GLRLM), 5.Gray Level Size Zone Matrix (GLSZM), 6.Gray Level Dependence Matrix (GLDM), 7.Neighborhood Gray-Level Transfer Differences Matrix (NGTDM).To standardize the scales of different features, normalization was applied using the maximum and minimum value normalization method, ensuring feature scales were between 0 and 1. Feature selection was performed using two methods to address the issue of strong feature correlation and prevent overfitting. Threshold: Features with variance below 0.1 were excluded, as these features showed slight variance and were less significant for sample differentiation.2.Least Absolute Shrinkage and Selection Operator (LASSO): This method was employed for dimensionality reduction by applying a penalty function (λ) to regulate variables. As λ increases, the coefficients of less impactful features are compressed to zero, allowing only the most significant features to remain. These methods were used to extract and screen features from enhanced CT and MRI images of HCC patients, selecting the most meaningful features for each imaging modality.

### Model construction and evaluation

Ninety-three HCC patients meeting the inclusion criteria were randomly assigned to either the training or validation group using the random number function in SPSS 26.0 software, with 7:3 ratio. The 66 patients in the training group were categorized into MVI-positive and MVI-negative groups based on pathological results. Comparative analysis was performed to identify differences in basic information and blood biochemical indexes between these groups.

One-way logistic regression analysis was used to screen for potential risk factors for MVI in HCC patients, followed by multi-factor logistic regression analysis to identify independent risk factors.

Model construction and evaluation in imagomics involved extracting CT and MRI features with variance thresholds above 0.1. Following Lasso regression analysis, enhanced CT and MRI radiomics models were developed using radiomics scoring formulas derived from variables with non-zero coefficients. A combined enhanced CT and MRI radiomics model was then established by performing Lasso regression on enhanced CT and MRI image features that exceeded a 0.1 variance threshold. The model’s calibration was assessed using validation group data, employing the area under the ROC curve (AUC) and the Hosmer-Lemeshow goodness of fit test to determine if the expected probability from clinical features aligned with the actual observed probability. The clinical benefit to patients was ultimately evaluated in depth using Decision Curve Analysis (DCA).

Basic patient data, independent risk factors identified through blood biochemical tests, and scores derived from the radiomics scoring formula via Lasso regression analysis were integrated. The diagnostic performance of the model was assessed using ROC analysis, calibration curves, and DCA. The validity and clinical relevance of the models were confirmed with the validation set data. By comparing their diagnostic efficiency, the most effective prediction model was determined.

### Statistical analysis

For the statistical analysis of clinical data and laboratory indicators, SPSS 26.0 software was utilized. The distribution of measurement data was tested using the Kolmogorov-Smirnov test to verify if it followed a normal distribution. Data not adhering to the normal distribution was presented using the median and interquartile range. The Mann-Whitney U test assessed differences between the training and verification groups’ negative groups. Qualitative parameters were denoted as the number of cases (percentage), and differences between the two groups were analyzed using either the chi-square test or the Fisher exact test, depending on the appropriateness of the data structure.

In radiomics, sketchable volumes of interest (VOIs) were delineated utilizing Python 3.10.1 software to extract radiomics features. These features were then subjected to variance selection, where those with a threshold value above 0.1 were retained for further analysis. The selected radiomics features underwent Lasso regression analysis, performed using the SPSSPRO 1.1.22 version, to validate their significance and utility. Moreover, the R 4.2.3 software was crucial in creating calibration curves and DCA. This software was also instrumental in evaluating the validity and reliability of all models through ROC AUC and the Hosmer-Lemeshow goodness of fit tests, ensuring a comprehensive analysis of the model’s performance and applicability.

## Results

### General baseline data analysis

Between October 2018 and September 2023, 207 patients with HCC underwent their initial partial hepatectomy at the Affiliated Hospital of Guilin Medical University. Following the application of predetermined inclusion and exclusion criteria, 93 of these patients were selected for a subsequent follow-up study. Seventy-eight patients had Barcelona Clinical liver cancer (BCLC) stage A, 11 had BCLC stage B, and 4 had BCLC stage C. These participants were then randomly assigned into two cohorts: a training group, consisting of 66 individuals, and a verification group, which comprised 27 individuals, adhering to a 7:3 division ratio. Within the training group, it was observed that 42 patients (representing 63.6% of the group) tested positive for MVI, while the remaining 24 (accounting for 36.4%) were MVI-negative. The verification group, on the other hand, included a total of 27 patients, among which 19 tested positive for MVI (making up 70.4% of this group), with the remaining 8 (or 29.6%) testing negative for MVI.

### General baseline data analysis results of the training group and validation group

Table 1 shows a comparative analysis of general clinical data between the training group and the verification group.

**Table 1:** Comparison of general clinical data between training group and verification group.

Variable	Total (n = 93)	Training group(n = 66)	Validation group (n = 27)	Statistic	P
Age(Year), Mean ± SD	54.89 ± 11.04	54.29 ± 11.28	56.37 ± 10.50	t = -0.824	0.412
ALB(U/L), Mean ± SD	38.42 ± 4.63	38.20 ± 4.77	38.96 ± 4.33	t = -0.711	0.479
PT(sec), Mean ± SD	11.82 ± 1.17	11.76 ± 1.16	11.98 ± 1.18	t = -0.818	0.415
TT(sec), Mean ± SD	18.44 ± 1.50	18.38 ± 1.52	18.58 ± 1.45	t = -0.587	0.559
LYMPH(10^9^/L), Mean ± SD	1.62 ± 0.57	1.66 ± 0.62	1.52 ± 0.45	t = 1.029	0.306
Diameter(mm), M (Q₁, Q₃)	60.00 (39.00–85.00)	60.00 (40.00–90.00)	60.00 (37.50–70.00)	Z = 0.882	0.378
CEA(ng/ml), M (Q₁, Q₃)	2.15 (1.47–3.31)	2.07 (1.44–3.19)	2.36 (1.56–3.44)	Z = 0.694	0.488
ALT(U/L), M (Q₁, Q₃)	29.30 (19.40–42.90)	29.74 (19.72–40.27)	27.90 (18.70–50.20)	Z = 0.351	0.725
AST(U/L), M (Q₁, Q₃)	34.70 (23.20–45.80)	35.85 (23.55–45.88)	33.20 (23.20–45.00)	Z = 0.669	0.504
TBIL(umol/L), M (Q₁, Q₃)	11.00 (8.13–14.70)	10.80 (7.36–13.39)	12.30 (9.45–18.25)	Z = 2.065	0.039
DBIL(umol/L), M (Q₁, Q₃)	4.87 (3.70–6.10)	4.85 (3.45–5.97)	4.87 (4.25–8.12)	Z = 1.410	0.159
r-GT(U/L), M (Q₁, Q₃)	70.00 (37.00–124.62)	70.50 (37.11–124.47)	69.00 (41.50–126.95)	Z = 0.135	0.892
ALP(U/L), M (Q₁, Q₃)	80.00 (64.00–106.00)	78.00 (64.25–97.75)	89.00 (63.00–121.00)	Z = 0.309	0.757
TP(U/L), M (Q₁, Q₃)	69.20 (64.97–73.66)	69.05 (64.12–73.35)	70.27 (66.96–75.62)	Z = 1.392	0.164
APTT(sec), M (Q₁, Q₃)	27.50 (25.00–31.10)	26.95 (24.63–30.78)	29.50 (26.35–31.60)	Z = 1.832	0.067
FIB(g/L), M (Q₁, Q₃)	2.57 (2.22–3.68)	2.69 (2.29–3.69)	2.45 (2.01–3.09)	Z = 1.282	0.200
PLT(10^9^/L), M (Q₁, Q₃)	187.00 (139.00–241.00)	205.00 (163.25–251.75)	158.00 (133.00–185.50)	Z = 2.357	0.018
NEUT(10^9^/L), M (Q₁, Q₃)	3.55 (2.55–4.83)	3.90 (2.89–5.29)	2.66 (2.13–3.54)	Z = 3.276	0.001
MVI, n (%)				χ^2^ = 0.385	0.535
negative	32 (34.41)	24 (36.36)	8 (29.63)		
positive	61 (65.59)	42 (63.64)	19 (70.37)		
Sex, n (%)				χ^2^ = 0.086	0.770
female	19 (20.43)	14 (21.21)	5 (18.52)		
male	74 (79.57)	52 (78.79)	22 (81.48)		
AFP(ng/ml), n (%)				χ^2^ = 0.156	0.692
<200	58 (62.37)	42 (63.64)	16 (59.26)		
≥200	35 (37.63)	24 (36.36)	11 (40.74)		
HBV DNA, n (%)				χ^2^ = 0.146	0.702
negative	25 (26.88)	17 (25.76)	8 (29.63)		
positive	68 (73.12)	49 (74.24)	19 (70.37)		
HBsAg, n (%)				χ^2^ = 0.291	0.590
negative	11 (11.83)	7 (10.61)	4 14.81)		
positive	82(88.17)	59 (89.39)	23 (85.19)		

Note: t: t-test; Z: Mann-Whitney test; SD: Standard deviation; M: Median, Q₁: 1st Quartile, Q₃: 3st Quartile; Z: Mann-Whitney test; χ^2^: Chi-square test; ALB: Albumin; AFP: Alpha-fetoprotein; ALT: Alanine aminotransferase; AST: Aspartate transferase; ALP, alkaline phosphatase; APTT: Activated partial thromboplastin time; CEA, Carcinoembryonic antigen; DBIL: Direct bilirubin; LYMPH: Lymph cell calculation; NEUT: Neutrophil count; mm: Millimeter; r-GT: R-glutamyltranspeptidase; TBIL: Total bilirubin; TT: Thrombin time; FIB: Fibrinogen TP: Total protein; PLT: Platelets; PT: Prothrombin time. P<0.05 was statistically significant.

### Logistics regression analysis

Logistics regression analysis results of MVI positive and MVI negative patients in the training group. The basic information and blood biochemical indexes of MVI-positive and MVI-negative patients in the training group were analyzed by single-factor analysis, and it was found that r-GT and maximum tumor diameter were statistically significant between MVI-positive and MVI-negative groups (p < 0.05). The multi-factor analysis found that the maximum tumor diameter was related to MVI, and the difference was statistically significant, as shown in Table 2.

**Table 2:** Univariate and multivariate results of MVI-positive and MVI-negative in training group.

Variables	Single factor					Multi-factor					
	β	S.E	Z	*p*	OR (95%CI)		β	S.E	Z	*p*	OR (95%CI)
Diameter	0.02	0.01	2.40	0.016	1.02 (1.01 ~ 1.04)		0.18	0.02	4.67	0.039	1.02 (1.01 ~ 1.04)
Age	-0.01	0.02	-0.62	0.537	0.99 (0.94 ~ 1.03)						
CEA	-0.19	0.14	-1.30	0.194	0.83 (0.63 ~ 1.10)						
ALT	0.02	0.01	1.51	0.130	1.02 (1.00 ~ 1.04)						
AST	0.02	0.01	1.51	0.131	1.02 (0.99 ~ 1.05)						
TBIL	0.05	0.05	1.16	0.247	1.06 (0.96 ~ 1.16)						
DBIL	0.15	0.11	1.41	0.159	1.16 (0.94 ~ 1.44)						
r-GT	0.01	0.00	2.08	0.038	1.01 (1.01 ~ 1.02)		0.01	0.00	1.77	0.077	1.01 (1.00 ~ 1.01)
ALP	0.01	0.01	1.41	0.158	1.01 (1.00 ~ 1.03)						
TP	0.01	0.03	0.44	0.661	1.01 (0.95 ~ 1.08)						
ALB	-0.08	0.06	-1.42	0.155	0.92 (0.82 ~ 1.03)						
PT	0.05	0.22	0.23	0.822	1.05 (0.68 ~ 1.63)						
APTT	-0.06	0.05	-1.16	0.246	0.94 (0.85 ~ 1.04)						
TT	0.06	0.17	0.36	0.718	1.06 (0.76 ~ 1.48)						
FIB	0.36	0.28	1.29	0.197	1.43 (0.83 ~ 2.47)						
PLT	0.00	0.00	0.69	0.489	1.00 (1.00 ~ 1.01)						
NEUT	0.01	0.09	0.11	0.914	1.01 (0.84 ~ 1.21)						
LYMPH	-0.00	0.42	-0.00	0.997	1.00 (0.44 ~ 2.27)						
Sex	0.45	0.66	0.68	0.497	1.56 (0.43 ~ 5.66)						
AFP	0.21	0.54	0.39	0.699	1.23 (0.43 ~ 3.53)						
HBV DNA	0.28	0.58	0.48	0.633	1.32 (0.43 ~ 4.08)						
HBsAg	0.31	0.81	0.38	0.706	1.36 (0.28 ~ 6.65)						

Note: CEA: Carcinoembryonic antigen; AFP: Alpha-fetoprotein; TBIL: Total bilirubin; DBIL: Direct bilirubin; TP: Total protein; ALB: Albumin; r-GT: R-glutamyltranspeptidase; ALT: Alanine aminotransferase; AST: Aspartate transferase; ALP, alkaline phosphatase; NEUT: Neutrophil count; LYMPH: Lymph cell calculation; PLT: Platelets; PT: Prothrombin time; APTT: Activated partial thromboplastin time; TT: Thrombin time; FIB: Fibrinogen; HBV DNA: Hepatitis B virus DNA; HBsAg: Hepatitis B surface antigen; OR value: Odds ratio value. P<0.05 was statistically significant.

### Radiomics feature screening results

The enhanced CT and MRI images of 93 HCC patients were included, and 2153 radiomics features were extracted by Python.

After variance selection, 14 radiomic features were screened by enhanced CT. After Lasso regression analysis, nine radiomic features with non-0 standardization coefficients were finally determined in the enhanced CT images of the training group, as shown in S1 Fig and Table 3. This information was incorporated into the construction of the enhanced CT radiomics prediction model, and finally, the enhanced CT radiomics feature model was obtained.

**Table 3:** Optimal radiomics feature of CT.

Variable name	Standardization coefficient	R^2^
Intercept	0.636	0.433
log-sigma-5-0-mm-3D_glszm_SmallAreaHighGrayLevelEmphasis	0.031	
wavelet-LHL_glszm_ZoneEntropy	0.03	
log-sigma-1-0-mm-3D_firstorder_Kurtosis	0.042	
wavelet-LLL_glszm_HighGrayLevelZoneEmphasis	0.046	
wavelet-HLL_firstorder_Range	0.016	
log-sigma-3-0-mm-3D_glrlm_LongRunHighGrayLevelEmphasis1	0.002	
log-sigma-5-0-mm-3D_glszm_SmallAreaHighGrayLevelEmphasis1	0.102	
log-sigma-2-0-mm-3D_glrlm_LongRunHighGrayLevelEmphasis1	0.055	
log-sigma-1-0-mm-3D_firstorder_Kurtosis1	0.029	

After variance selection, 18 radiomics feature indicators with a threshold value greater than 0.1 were obtained in the training group’s MRI images. Finally, after Lasso analysis, five radiomics features with a non-0 normalization coefficient were determined and included in the construction of the predictive model of the training group’s MRI omics, as shown in S2 Fig and Table 4.

**Table 4:** Optimal radiomics feature of MRI.

Variable name	Standardization coefficient	R^2^
Intercept	0.636	0.425
square_firstorder_Range1M	0.009	
wavelet-HLL_glszm_ZoneEntropy1M	0.125	
wavelet-LHH_gldm_LargeDependenceLowGrayLevelEmphasis1M	0.037	
square_firstorder_KurtosisM	-0.03	
wavelet-HLH_glszm_HighGrayLevelZoneEmphasisM	-0.022	

Lasso analysis was carried out by combining the non-0 radiomic features with enhanced CT and MRI, and an radiomic model of CT combined with MRI was established, as shown in S3 Fig and Table 5.

**Table 5:** Optimal radiomics features of CT combined with MRI.

Variable name	Standardization coefficient	R^2^
Intercept	0.636	0.559
wavelet-HLL_firstorder_Range	0.003	
square_firstorder_Range1M	0.007	
wavelet-HLL_glszm_ZoneEntropy1M	0.098	
wavelet-LHH_gldm_LargeDependenceLowGrayLevelEmphasis1M	0.026	
square_firstorder_KurtosisM	-0.044	
wavelet-HLH_glszm_HighGrayLevelZoneEmphasisM	-0.02	
log-sigma-1-0-mm-3D_firstorder_Kurtosis	0.021	
log-sigma-5-0-mm-3D_glszm_SmallAreaHighGrayLevelEmphasis1	0.085	
log-sigma-5-0-mm-3D_glszm_SmallAreaHighGrayLevelEmphasis	0.009	
wavelet-LLL_glszm_HighGrayLevelZoneEmphasis	0.03	

### The efficiency of the radiomics prediction model in predicting MVI

**Fig 2:**
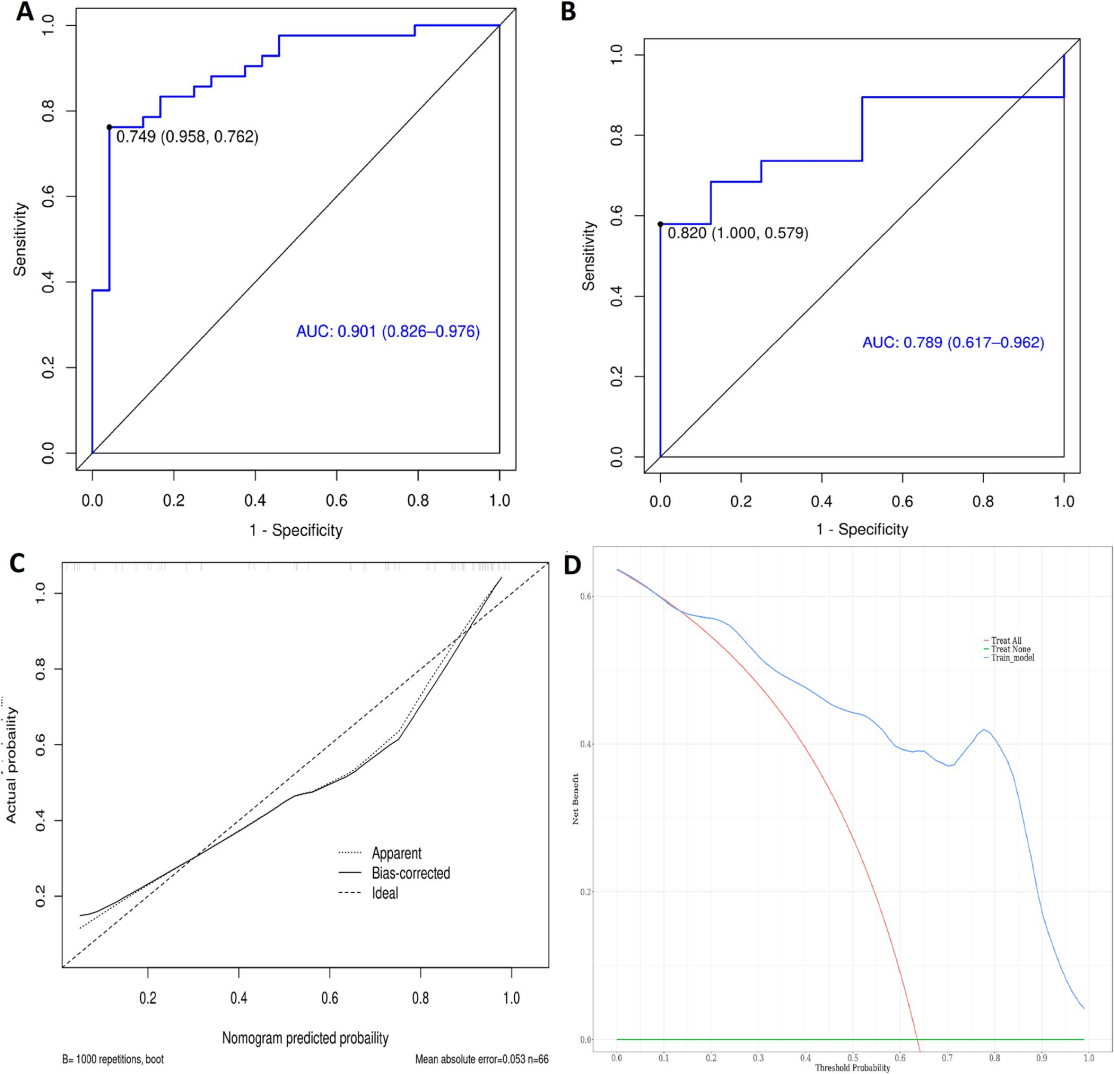
The ROC, calibration curve and DCA of the predictive model based on enhanced CT. **(**A) The ROC graph of the training group. (B) The ROC graph of the verification group. (C) The calibration curve of the training group, χ^2^ = 12.408, P = 0.1339. (D) DCA curve of training group.

**Fig 3:**
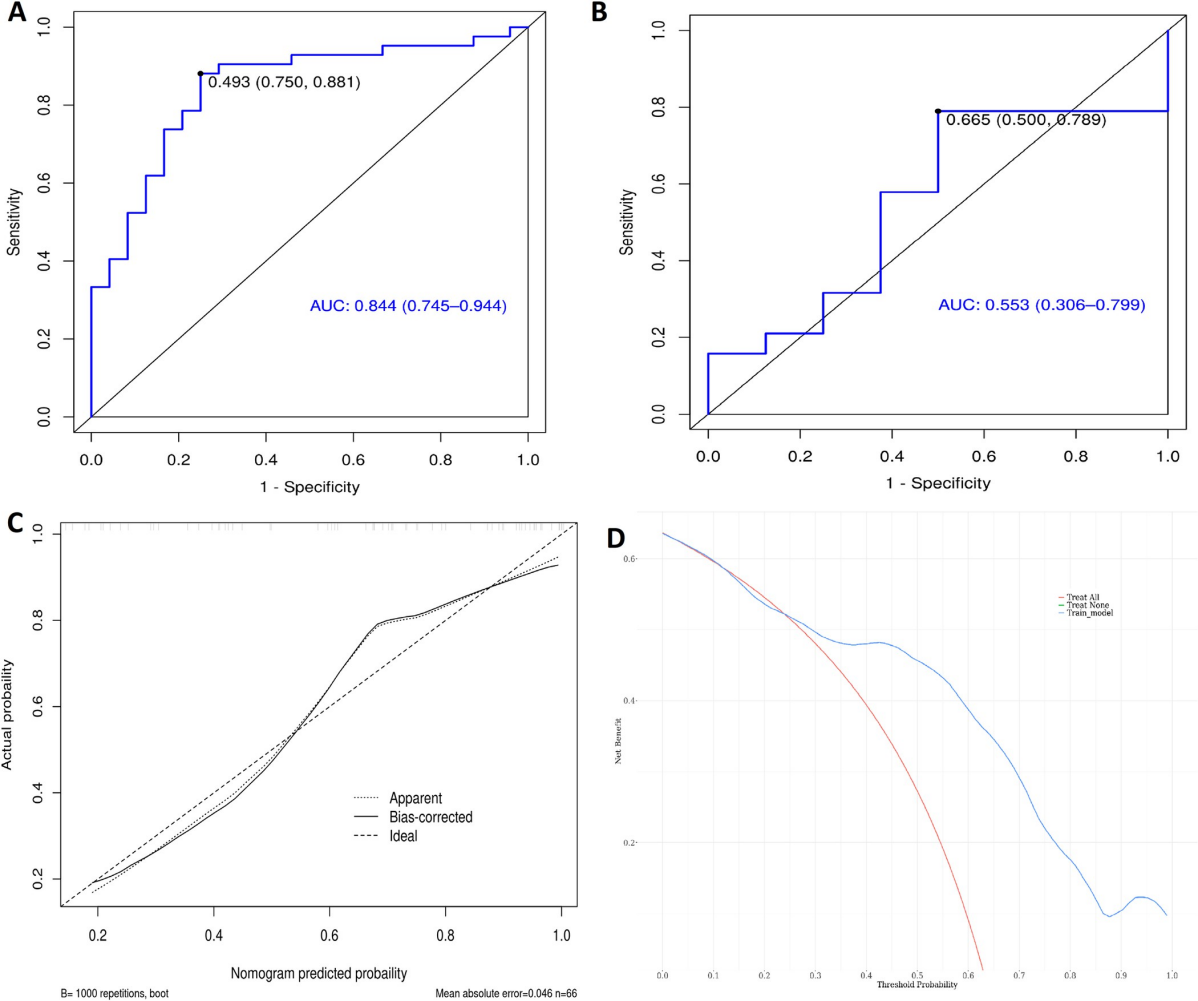
The ROC, calibration curve and DCA of the prediction model based on MRI. (A) The ROC graph of the training group. (B) The ROC graph of the verification group. (C) The calibration curve of the training group,χ^2^ = 8.0183, P = 0.4317. (D) DCA curve of training group.

Based on the selected CT radiomics characteristics, a CT radiomics prediction model was established. The effectiveness of the model was evaluated using AUC, the calibration curve, and the DCA curve, as shown in Fig 2. A predictive model was established based on the characteristics of the selected MRI radiomics. The model’s effectiveness was evaluated by AUC, calibration curve, and DCA curve, as shown in Fig 3.

**Fig 4:**
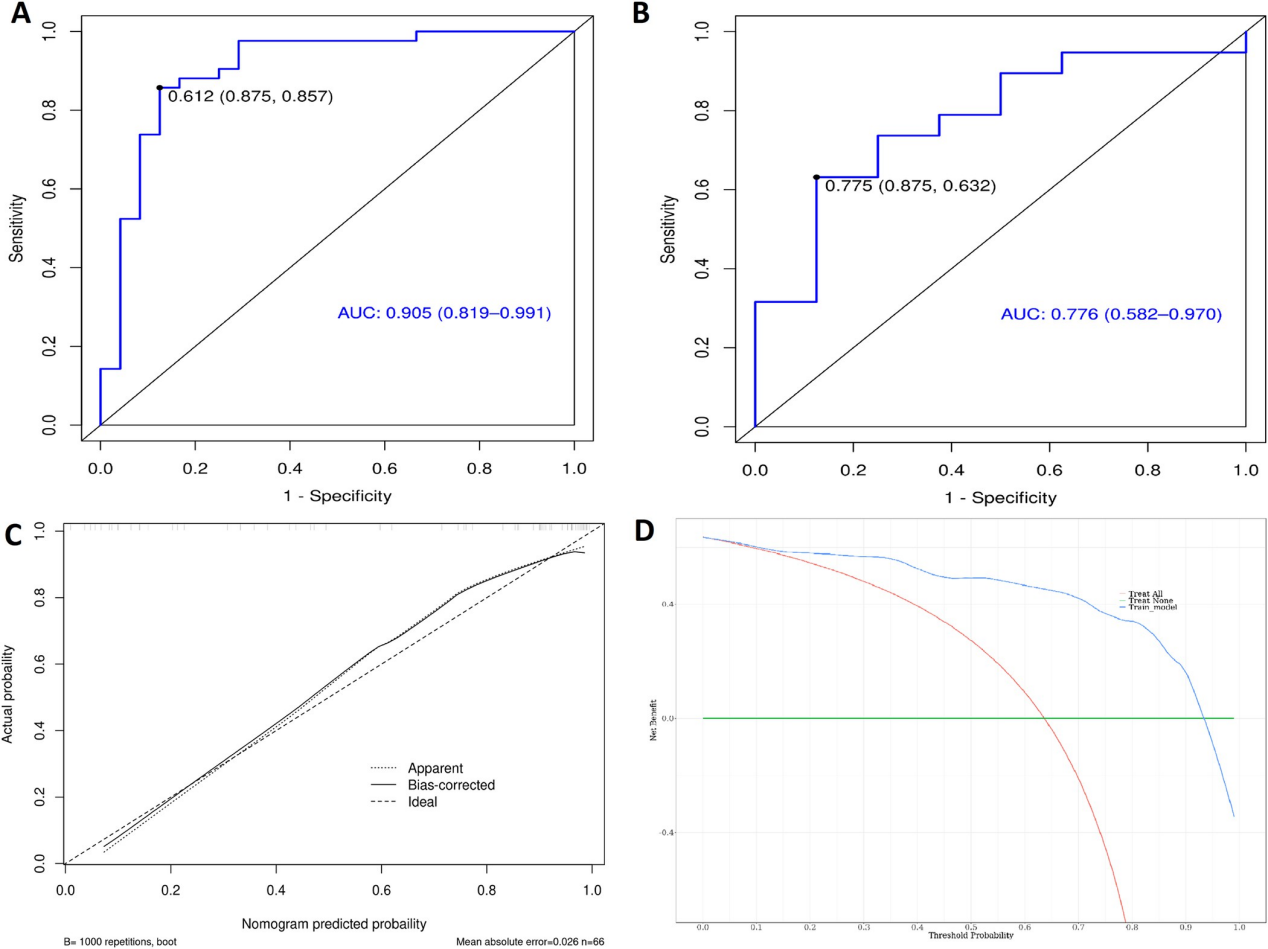
The ROC calibration curve and DCA of the combined prediction model based on CT and MRI. **(**A) The ROC graph of the training group. (B) The ROC graph of the verification group. (C) The calibration curve of the training group, χ^2^ = 12.064, p = 0.1484. (D) DCA curve of training group.

The diagnostic performance of a single index prediction model is not high, so we set up a comprehensive prediction model of multiple indicators. Firstly, the combined radiomics prediction model of the CT and MRI was established in this study, and its diagnostic efficacy was evaluated in Fig 4. The area AUC under the ROC curve of the prediction model for MVI in the training group was 0.905, 95%CI (0.819–0.991), and sensitivity and specificity were 85.7% and 87.5%. The area AUC under the ROC curve of the prediction model for MVI in the verification group was 0.776, 95%CI (0.582–0.970), and sensitivity and specificity were 63.2% and 87.5%.

**Fig 5:**
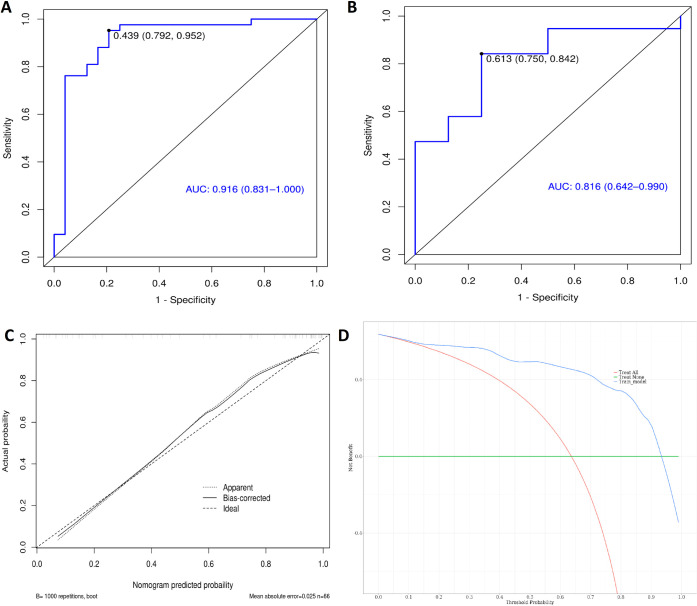
The ROC, calibration curve and DCA of the combined prediction model based on tumor diameter combined with the optimal radiomics features. (A) The ROC graph of the training group. (B) The ROC graph of the verification group. (C) The calibration curve of the training group, χ^2^ = 12.064, p = 0.1484. (D) DCA curve of training group.

Finally, we established a predictive model of tumor diameter combined with CT and MRI omics features (Fig 5). The AUC of the prediction model for the diagnosis of MVI in the training group was 0.916, 95%CI (0.843–1.000), and sensitivity and specificity were 95.2% and 79.2%, respectively. In the verification group, the AUC of the prediction model for the diagnosis of MVI was 0.816, 95% CI: (0.642–0.990), and sensitivity and specificity were 84.2% and 75.0%, respectively. Regarding fitting degrees, the MAE of the training group was 0.025. The DCA curve of the training group can be concluded that when the threshold value is more significant than 0.15, the DCA curve is above None and All, indicating that the effect of the model is acceptable within this range. The combined model of three indexes is better than the model of other single indexes or the combined model of two indexes, as shown in S1 Table.

## Discussion

HCC is a highly aggressive malignancy, and its MVI status has an important impact on the prognosis and treatment options of patients [10,11]. However, accurate prediction of MVI in HCC patients has been a major clinical challenge. Traditional prediction methods mainly rely on the imaging morphological characteristics of patients, such as judging MVI according to tumor size, envelope integrity, tumor surface smoothness, low peritumoral signal, and other imaging characteristics [12]. Although it has a certain reference value, its accuracy and reliability are often questioned. These methods usually fail to fully reflect the complexity and heterogeneity within tumors, resulting in inaccurate prediction results. In recent years, with the emergence of radiomics, it can better transform the inherent heterogeneity of tumors into digital signals [13]. Through detailed analysis of tumor images, we can extract a large number of data related to tumor features, such as texture and morphological features [14]. These data can more comprehensively reflect tumors’ internal structure and biological characteristics, thus providing us with a more accurate basis for prediction [15]. In recent years, some studies have successfully discovered the radiomics features related to MVI based on enhanced CT or MRI radiomics technology and established an omics model for preoperative prediction [16]. These models could accurately predict MVI in patients with HCC by comprehensively analyzing image-omics features. The study showed that these models performed well in predicting MVI in patients with HCC, providing us with a new perspective to assess MVI risk in patients with HCC.

In terms of maximum tumor diameter, one study found that tumor size in HCC patients was correlated with the incidence of MVI [10]. Moreover, Chen’s [17] study also found the same conclusion as ours, that tumor size was related to MVI. The ROC AUC of the tumor diameter model is more significant than 0.7 in both the training and verification groups, and the calibration curve results fit well with the diagonal dotted line Idea, indicating that the model has a good prediction effect. However, the threshold of the DCA curve in the training group and the verification group is relatively large. This indicates that the clinical applicability of the drug is relatively poor. It is possible that the larger the tumor, the greater the chance that the cancer will involve blood vessels and the higher the chance of MVI.

In their study, Feng et al. extracted radiomics features from enhanced CT of HCC patients before surgery and found that the texture features had a high correlation with MVI, providing a new perspective for predicting MVI of HCC [18]. The AUC of the 11 optimal radiomics features selected by the CT radiomics model was 0.84 in the imaging model verification group and 0.80 in the training group [18]. According to the results of LU’s [19] study on the prediction of MVI by Gd-EOB-DTPA enhanced MRI, seven radiomics features were extracted, and the prediction model was established. The AUC of the predictive model in the training group was 0.826. Lei’s [20] results showed that the multiple models based on enhanced CT and MRI enhanced deep learning (Enhanced CT was combined with enhanced MRI) model of the AUC was 0.844, which was better than that of single phase as CT prediction model (AUC = 0.706 0.776, P < 0.05), or single sequence MRI prediction model (AUC = 0.706–0.717, p < 0.05). To establish a more comprehensive prediction model, we established a prediction model with comprehensive tumor diameter and radiomics features of CTand MRI. The ROC AUC of the model was more significant than 0.9 in the training group and superior to the previous AUC of single radiomics features of CT or MRI, or both. The results of the calibration curve fit well with the diagonal dashed Idea. It showed that the prediction effect of the model was good. For patients with MVI, enlarge surgical margin during surgery could effectively reduce postoperative HCC recurrence [5]. Therefore, effectively predicting the MVI condition of patients before surgery was conducive to making surgical decisions [21].

### Lack of research

Although the results of this study are satisfactory, there were still some potential limitations. 1. This study was a single-center retrospective design with limited cases. 2. Regarding data acquisition and processing, we adopted the 3D manual segmentation of ROI to obtain radiomics features. The quality of manual segmentation of ROI was affected by the operator’s technical level, which may lead to the deterioration of the stability of the experimental results. 3. Radiomics features of hepatobiliary stage were not extracted. Future multi-center, big data, forward-looking design, and more reliable and accurate automatic or semi-automatic segmentation methods are needed.

## Conclusion

The joint model that integrated the optimal radiomics features with clinical variable has good diagnostic performance for predicting microvascular invasion in hepatocellular carcinoma patients and certain clinical applicability.

## Supporting information

S1 Fig Cross-validation of Lasso regression of enhanced CT images. Binomial deviation and log (lambda) drawing of enhanced CT image features in training group in LASSO model screening The y-axis represents the binomial deviation and the x-axis represents the average number of predictors on log (lambda) Draw a solid line at the value with the minimum and the minimum 1 error The penalty parameter lambda is selected by cross-validation based on the minimum 1000 times λ = 0.061 log (λ) = -2.803.
(TIF)

S2 Fig Cross-validation of Lasso regression of enhanced MRI images. Binomial bias and log (lambda) rendering in LASSO model screening of the features of Gd-EOB-DTPA MRI images in training group The y-axis represents the binomial deviation and the x-axis represents the average number of predictors on log (lambda) Draw a solid line at the value with the minimum and the minimum 1 error The penalty parameter lambda is selected by cross-validation based on the minimum 1000 times λ = 0.101 log (λ) = -2.293.
(TIF)

S3 Fig The cross-validation diagram of Lasso regression of enhanced CT combined with MRI images. Binomial bias and log (lambda) rendering in LASSO model screening of radiomics features of enhanced CT and Gd-EOB-DTPA MRI in training group The y-axis represents the binomial deviation and the x-axis represents the average number of predictors on log (lambda) Draw a solid line at the value with the minimum and the minimum 1 error The penalty parameter lambda is selected by cross-validation based on the minimum 1000 times λ = 0.081 log (λ) = 2.516.
(TIF)

S1 Table ROC calibration curves and DCA results of different models. Model 1: Tumor diameter; Model 2: CT; Model 3: MRI; Model 4::CT+ MRI; Model 5: Tumor diameter + CT: Model 6: Tumor diameter + MRI; Model 7: Tumor diameter + CT + MRI.
(DOCX)

S1 File Statistically relevant information. (ZIP)

## Abbreviations

AFP: Alphaf etoprotein
ALB: Serum albumin
ALP: Alkaline Phosphatase
ALT: Alanine transaminase
APTT: Activated partial thromboplastin time
AST: Aspartate transaminase
BCLC: The Barcelona-Clnic Liver Cancer
CEA: Carcinoembryonic antigen
DBIL: direct bilirubin
FIB: Fibrinogen
HCC: Hepatocellular carcinoma
MVI: Microvascular invasion
PLC: Primary liver cancer
PLT: Platelet
PT: Prothrombin time
ROI: region of interest
TB: Total bilirubin,TP: Total Protein
TT: Thrombin time
