# Supplementary figures and images for: A clinical study exploring the prediction of microvascular invasion in hepatocellular carcinoma through the use of combined enhanced CT and MRI radiomics
Source: PLoS One. 2025 Jan 28;20(1):e0318232. doi: 10.1371/journal.pone.0318232 (PMC11774365; doi:10.1371/journal.pone.0318232)

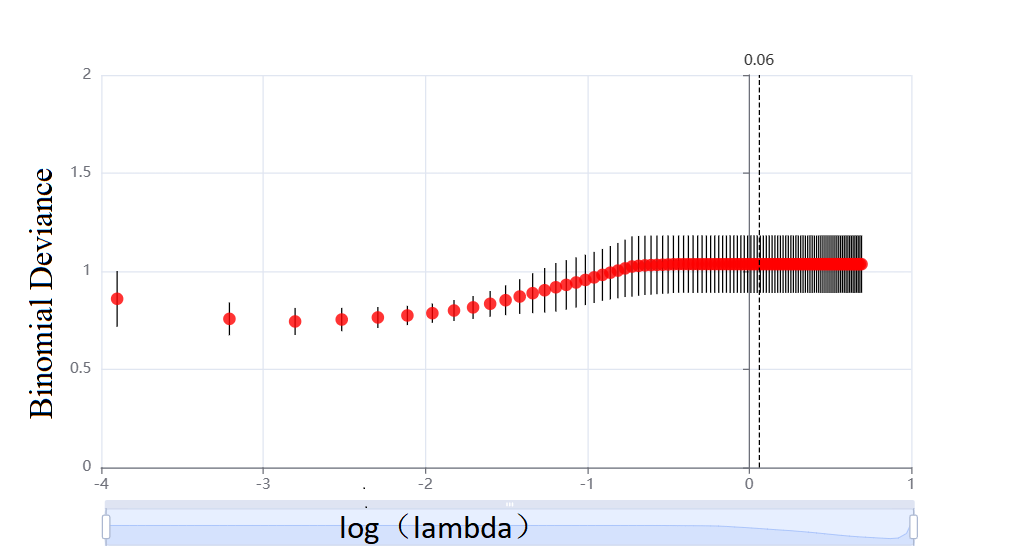

Supplement: S1 Fig — Binomial deviation and log (lambda) drawing of enhanced CT image features in training group in LASSO model screening The y-axis represents the binomial deviation and the x-axis represents the average number of predictors on log (lambda) Draw a solid line at the value with the minimum and the minimum 1 error The penalty parameter lambda is selected by cross-validation based on the minimum 1000 times λ = 0.061 log (λ) = -2.803. (TIF) [file pone.0318232.s001.tif]

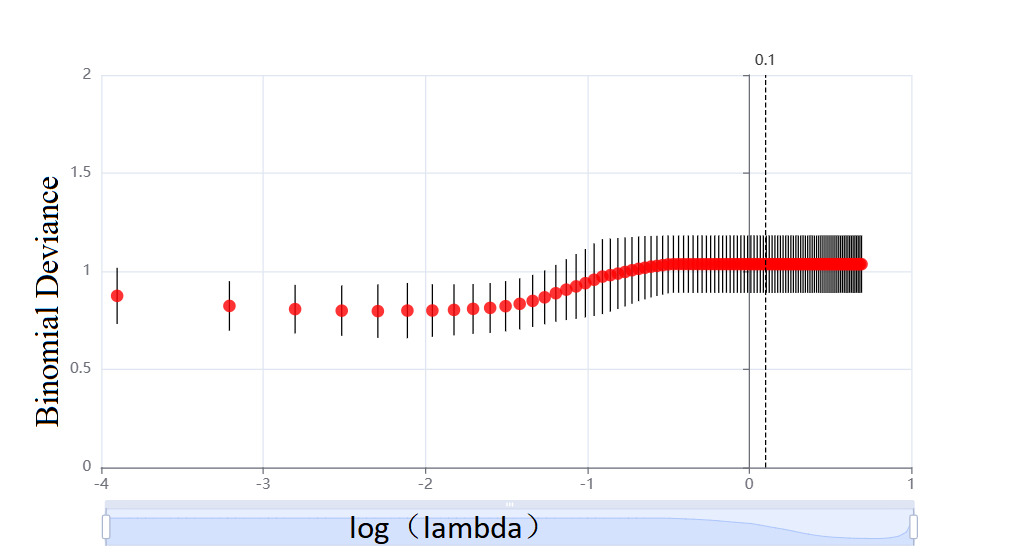

Supplement: S2 Fig — Binomial bias and log (lambda) rendering in LASSO model screening of the features of Gd-EOB-DTPA MRI images in training group The y-axis represents the binomial deviation and the x-axis represents the average number of predictors on log (lambda) Draw a solid line at the value with the minimum and the minimum 1 error The penalty parameter lambda is selected by cross-validation based on the minimum 1000 times λ = 0.101 log (λ) = -2.293. (TIF) [file pone.0318232.s002.tif]

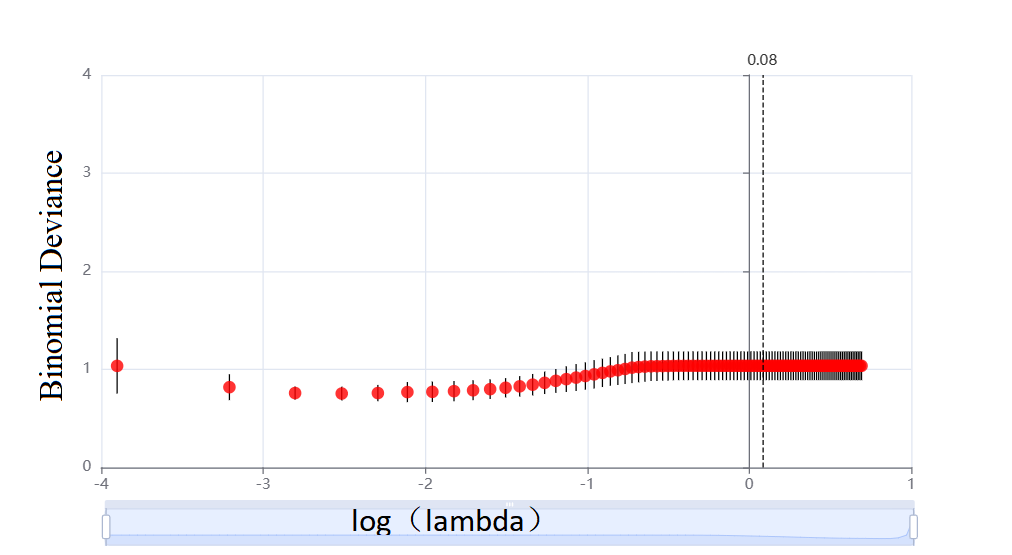

Supplement: S3 Fig — Binomial bias and log (lambda) rendering in LASSO model screening of radiomics features of enhanced CT and Gd-EOB-DTPA MRI in training group The y-axis represents the binomial deviation and the x-axis represents the average number of predictors on log (lambda) Draw a solid line at the value with the minimum and the minimum 1 error The penalty parameter lambda is selected by cross-validation based on the minimum 1000 times λ = 0.081 log (λ) = 2.516. (TIF) [file pone.0318232.s003.tif]
