# Supplementary material for: A clinical study exploring the prediction of microvascular invasion in hepatocellular carcinoma through the use of combined enhanced CT and MRI radiomics
Source: PLoS One. 2025 Jan 28;20(1):e0318232. doi: 10.1371/journal.pone.0318232 (PMC11774365; doi:10.1371/journal.pone.0318232)
Supplement: S1 Table — Model 1: Tumor diameter; Model 2: CT; Model 3: MRI; Model 4::CT+ MRI; Model 5: Tumor diameter + CT: Model 6: Tumor diameter + MRI; Model 7: Tumor diameter + CT + MRI. (DOCX) [file pone.0318232.s004.docx]

| Table S1 ROC calibration curves and DCA results of different models |
| --- |
| \| models \| ROC \| \| \| \| \| \| DCA \|  \| \| --- \| --- \| --- \| --- \| --- \| --- \| --- \| --- \| --- \| \| Training group \| \| \| Verification group \| \| \| Training group \| Training group \| \| sensitivity \| specificity \| ROC \| sensitivity \| specificity \| ROC \| Diagonal dashed line Ideal fit degree \| Benefit threshold \| \| Model 1 \| 0.716 \| 0.667 \| 0.716 \| 0.737 \| 0.750 \| 0.753 \| Relatively good \| 0.40 \| \| Model 2 \| 0.762 \| 0.958 \| 0.901 \| 0.579 \| 1.000 \| 0.842 \| Relatively good \| 0.15 \| \| Model 3 \| 0.881 \| 0.750 \| 0.884 \| 0.789 \| 0.500 \| 0.553 \| Relatively good \| 0.25 \| \| Model 4 \| 0.857 \| 0.875 \| 0.905 \| 0.632 \| 0.875 \| 0.776 \| Relatively good \| 0.15 \| \| Model 5 \| 0.833 \| 0.917 \| 0.905 \| 0.526 \| 1.000 \| 0.796 \| Relatively good \| 0.15 \| \| Model 6 \| 0.881 \| 0.792 \| 0.854 \| 0.737 \| 0.750 \| 0.763 \| Rrelatively good \| 0.25 \| \| Model 7 \| 0.972 \| 0.952 \| 0.916 \| 0.952 \| 0.792 \| 0.816 \| Relatively good \| 0.15 \| |

Note：Model 1：Tumor diameter； Model 2：Enhanced CT；Model 3：Gd-EOB-DTPA enhanced MRI；Model 4：：Enhanced CT+ Gd-EOB-DTPA enhanced MRI； Model 5：Tumor diameter + Enhanced CT：Model 6：Tumor diameter + Gd-EOB-DTPA enhanced MRI；Model 7：Tumor diameter + Enhanced CT + Gd-EOB-DTPA enhanced MRI
